# Experimental study of seepage-scour failure in geotextile tubes gap with damaged vertical sidewall

**DOI:** 10.1371/journal.pone.0352901

**Published:** 2026-07-28

**Authors:** Wen-Long Mao, Ling Zhang, Tian-Wen Wang

**Affiliations:** 1 School of Naval Architecture & Intelligent Manufacturing, Jiangsu Maritime Institute, Nanjing, P. R. China; 2 College of Biosystems Engineering and Food Science, Zhejiang Univ, Hangzhou, P. R China; Ardakan University, IRAN, ISLAMIC REPUBLIC OF

## Abstract

Geotextile tubes, hydraulically filled with a slurry of fine silt and water, have been variously applied in hydraulic and coastal engineering fields. However, geotextile damage poses a great threat to structures made of geotextile tubes. When a water head difference exists across the tube, the soil in damaged tubes is affected by the dual actions of seepage and scour. To investigate soil failure patterns and tendencies of damaged tubes under hydraulic action, a structural apparatus and the corresponding test method were designed. Four factors considered were the radius of the damaged area (*r*_*0*_: 0.25–2.0 cm), the grain size distribution (Sand B C_*u*_ = 3.4, Sand E C_*u*_ = 50), the scouring flow velocity (*v*: 0–4 cm/s), and the hydraulic gradient. The results showed that the scouring flow exerted a limited effect on the failure mode of sand in the tubes, and that the failure process of sand in the tubes could be divided into three stages including a stable, an initial erosion, and a cyclic sand outflow stage. The hydraulic gradient at the initial erosion stage was defined as the critical gradient(*j*_*cr*_), which was interactively influenced by sand gradation, damage radius, and scouring flow velocity. Under identical conditions, Sand E exhibited a higher resistance against seepage-induced failure than Sand B. In terms of stability under varying conditions, for Sand B, increasing the damage radius (tested at flow velocities of 0–4 cm/s) reduced *j*_*cr*_ by 94%–100%, while increasing the flow velocity (tested at damage radii of 0.25–2.0 cm) reduced *j*_*cr*_ by 60%–100%. For Sand E, the corresponding reductions were 83%–95% and 58%–88%, respectively, further confirming Sand E’s superior erosion resistance.

## 1. Introduction

Geotextile tube technology has the advantages of low carbon emissions, energy savings, simple processing, low cost, and a controllable construction period [1,2], rendering it widely applied in the construction of various projects such as estuary reservoir dams for freshwater storage, coastal beach reclamation, port quay cofferdams, and waterway regulation [3,4].

The sand contained in tubes rarely leaks directly through the geotextiles which meet the basic requirements of drainage and soil conservation. However, the geotextile damage occurs occasionally due to the potential bursting, puncturing, wearing, and abrasion caused by sharp and large particles in the filling slurry as well as improper operation by construction personnel [5,6]. In addition, animal burrowing and sand or gravel waves driven by turbid flows during construction and operation may cause an aggravation of geotextile damage [7].

**Fig 1:**
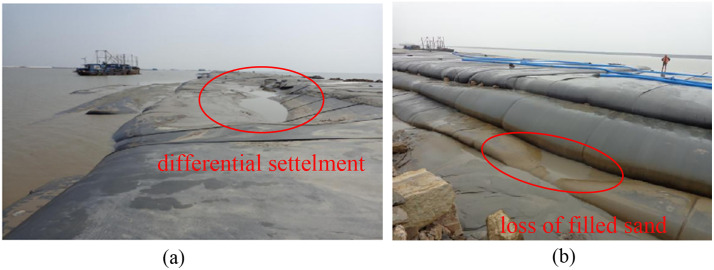
Photos of differential settlement and sand loss.

Once the geotextile is damaged, the sand in the tubes may undergo seepage failure under the condition of the hydraulic head difference through the structure. When damage occurs at the joints between adjacent tubes, the exposed sand within the damaged zone becomes susceptible to further erosion, leading to more severe seepage failure. A case in point is the Tianjin South Port project, where embankments constructed with sand-filled geotextile tubes experienced loss of filled sand and differential settlement during construction (as shown in the Fig 1).

Even completed structures are not immune to such risks. For instance, the geotextile tube dam at the Chenhang Reservoir in the Yangtze Estuary, Shanghai, has developed significant subsidence at one corner after decades of service. Direct observation and analysis indicate that these failures are primarily attributed to the substantial loss of soil particles through the seams between geotextile tubes [8]. Specifically, seepage forces induce the outflow of sand from the tube interior via any damaged hole. This transported material is then further carried beyond the dam structure by the combined action of seepage and the scouring flow within the joint channels, ultimately triggering dam collapse.

Although seepage failure caused by geotextile damage, which may lead to dam failure or collapse, has been observed in some engineering projects [9], research about the filtration effect and seepage characteristics of damaged geotextile tube still remains lacking because of the hidden nature of geotextile damage and the delayed consequences.

Regarding the issue of geotextile damage, Bolt [10] investigated the impact of damage shapes (I, L, and O-shaped) on filtration performance. The results indicated that the O-shaped damage had the poorest ability to prevent seepage deformation under variable hydraulic head conditions. Chew et al. [11] conducted a series of tests using geotextile specimens with pre-cut L-shaped holes of varying sizes to simulate punctures caused by installation damage. They found that geotextiles with holes up to a certain critical size could still perform satisfactorily if soil arching was fully developed. The critical size of the pre-cut hole was determined to be a function of the geotextile’s properties, wave period and cycles of wave load applied onto the soil. Apart from these findings, there exists no further literature addressing the filtration characteristics following damage to geotextiles. Many researchers have focused on the mechanical and hydraulic properties of geotextiles.

In the field of seepage failure in soil mechanics, Qin et al. [12] investigated patterns of soil loss and deformation induced by tunnel leakage, examining the influence of initial fine particle content, soil internal friction angle, and tunnel opening location on both leakage dynamics and ground settlement. Wang et al. [13] investigated the efficacy of solidified soil in scour protection, focusing on three key factors: cured strength, curing state, and the extent of solidification. The results demonstrated that optimal cured strength and state can significantly mitigate scour. Zhang et al. [14] conducted model tests on backward erosion piping under a K0 stress state, revealing that the piping process in gap-graded soils can be divided into three stages: seepage, pipe formation, and pipe wall erosion. Their study highlighted the influence of grain size ratio, hydraulic head, and fine content on erosion behavior, and demonstrated that the transition from suffusion to piping depends on critical thresholds of these parameters.

In terms of experimental apparatus, recent studies have developed specialized permeameters to investigate soil suffusion under controlled conditions. Chen et al. [15] designed a large-sized permeameter to study suffusion characteristics of anisotropic soils under controlled hydraulic gradients and stress states. Chen et al. [16] also developed a new flexible-wall triaxial permeameter that enables localized characterization of soil suffusion. Despite these valuable contributions from the broader soil mechanics community [17], it is difficult to apply the relevant conclusions to seepage failure resulting from damaged geotextiles. This is because geotextiles, functioning as filtration materials, are distinctly different from soil filtration layers.

**Fig 2:**
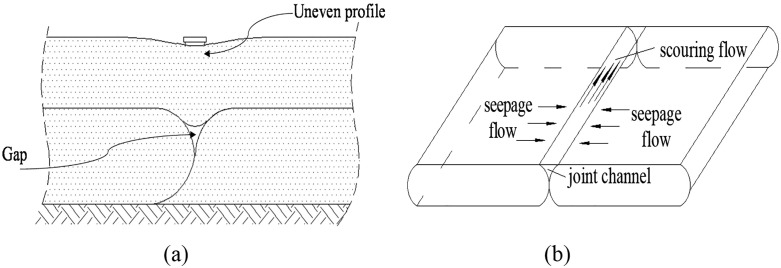
Joint between adjacent geotextile tubes and the formation of scouring flow in a joint channel. (a) Joint between adjacent geotextile tubes, (b) formation of scouring flow.

On the other hand, the water that permeating through the tubes would gather within the joint channels (called gaps, as shown in Fig 2 (a) [18]) between adjacent tubes, thereby generating a scouring flow, as shown in Fig 2(b). The sand generated by seepage failure migrates with the scouring flow in the joint channels, which further exacerbating seepage failure. To address the issue of lap joints between adjacent tubes, a novel geotextile tube connection method was developed, and field verification showing its effectiveness were run [18]. However, this technology has not been fully promoted. Notably, appropriate measures have not been implemented at the joints between adjacent geotextile tubes in many earlier projects, and the corresponding engineering issues have not attracted notably attention from academia.

In essence, the damage to the sidewalls of joint channels in geotextile tube dams can be attributed to the combination of seepage deformation and flow scouring along joint channels. Some soil erosion studies have noted the correspondence between the sediment incipient motion and flow velocity [19,20]. Guo et al. [21] proposed a coarse-graining CFD-DEM model to study contact erosion at coarse/fine soil interfaces, quantifying the corresponding flow discharge rate within the fine soil fraction. Wu et al. [22] investigated the incipient motion velocity of non-uniform sediment particles incorporating the additional mass effect through combined mechanical analysis and experimental validation. Liu et al. [23] performed comprehensive experimental investigations to examine sediment incipient motion across varying relative density conditions on different seabed types. Their study incorporated internal friction force as a key parameter to characterize relative density effects, ultimately establishing a unified velocity function for sediment initiation. Although these studies have examined various factors affecting sediment initiation, they have not taken into account the effect of seepage erosion. Therefore, the issues caused by damage to geotextiles in the joints of geotextile tubes still need to be studied in depth.

Research has shown that horizontal seepage failure causes more severe damage to the sand structure than vertical upward seepage failure does [24,25]. Therefore, the present study focused on analyzing horizontal seepage and scour failure when damage to sidewalls occurs. Four variables including grain size distribution, radius of the circular damaged hole, scouring flow velocity and hydraulic gradient were selected. The main purpose of this study was to investigate the combined effect of seepage and scouring flow at the joints of geotextile tubes with damaged sidewalls and the migration pattern of sand in the joint channels between geotextile tubes. The specific objectives of this study were to: (1) develop a set of model test device and method that characterizes the real conditions caused by geotextile tube damage; (2) conduct a series of tests to obtain the patterns and tendencies of erosion; (3) calculate the critical gradient of erosion failure; and (4) evaluate the effects of sand gradation and scouring flow on the structural safety. These findings of this study can provide valuable references for the application of geotextile tubes and geotextile bags in engineering, as well as for addressing related seepage and scouring issues.

## 2. Materials and methods

### 2.1. Test apparatus and materials

**Fig 3:**
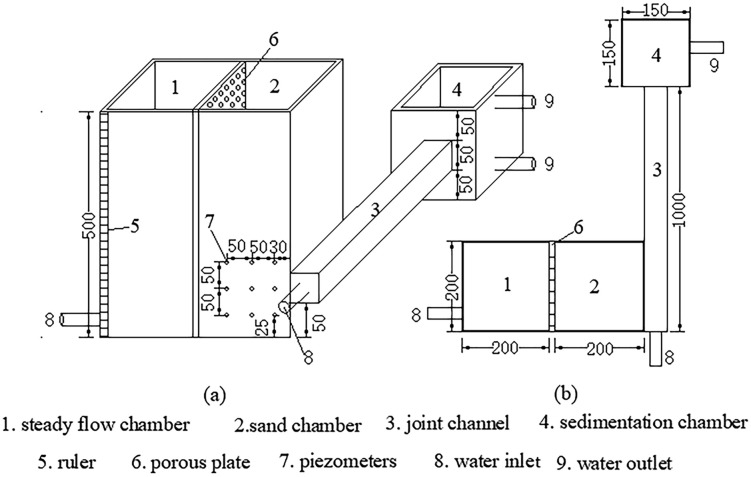
Test apparatus for seepage and scour failure at the joint between tubes (unit: mm). (a) Axonometric view. (b) top view.

The test apparatus comprises a steady flow chamber, a sand chamber, a joint channel, and a sedimentation chamber, as shown in Fig 3. All the chambers were made of transparent acrylic so that seepage and scouring progress can be observed in the tests. The steady flow chamber, with dimensions of 200 × 200 × 500 mm, aims to simulate the upstream water level while reducing the impact of turbulence. A ruler is attached to the sidewall to measure the upstream head. An opening on the left side is connected to a liftable seepage flow water tank, which provides an upstream seepage head during the test. The sand chamber with dimensions of 200 × 200 × 500 mm aims to simulate a tube, in which the sand subjected to seepage. The junction between the sand chamber and the steady flow chamber is a porous acrylic plate covered with a geotextile. A horizontal seepage test revealed that the overburden pressure does not affect the seepage failure gradient [24]. To better simulate the horizontal flow in the seepage process, the top of the sand chamber was sealed.

The channel simulated the joint between adjacent tubes, which in practice can exhibit various cross-sectional geometries (e.g., rhombic, triangular, or rectangular) with a diameter of approximately 50 mm. Since such complex geometries are difficult to control in physical experiments, following the simplified channel approach used by Man et al. [26–28], a rectangular channel with dimensions of 50 × 50 × 1000 mm was adopted as a well-defined representative to investigate the fundamental mechanism of sand failure induced by geotextile damage and head difference. The inner side of the channel connected to the sand chamber was covered with a geotextile with a circular damage. The upper, lower, and outer sides of the channel were not modified to facilitate observation. The end of the channel is connected to a sedimentation chamber with dimensions of 150 × 150 × 150 mm. The sedimentation chamber is open at the top and contains an outlet with a radius of 5 mm in the upper and lower parts of one side. The lower outlet was used to quickly drain water from the apparatus after the test, and the upper outlet maintained a constant downstream water level during the test.

The channel length in the apparatus is significantly shorter results in a much smaller cross-sectional area for seepage flow compared to that in the gaps between tubes in actual projects. Relying solely on seepage inside the apparatus, the flow rate and velocity in the channel could be far lower than those in actual joints. Therefore, a water inlet for scouring flow with a radius of 5 mm was placed at the upstream end of the channel and connected to another liftable scouring flow water tank. In the test, this water tank was lifted to increase the flow rate in the channel to simulate the scour effect in actual projects as closely as possible. The flow velocity in the channel was monitored via an infrared flow velocity measuring instrument, with a measurement range of 0–10 cm/s and an accuracy of 0.1 cm/s.

**Fig 4:**
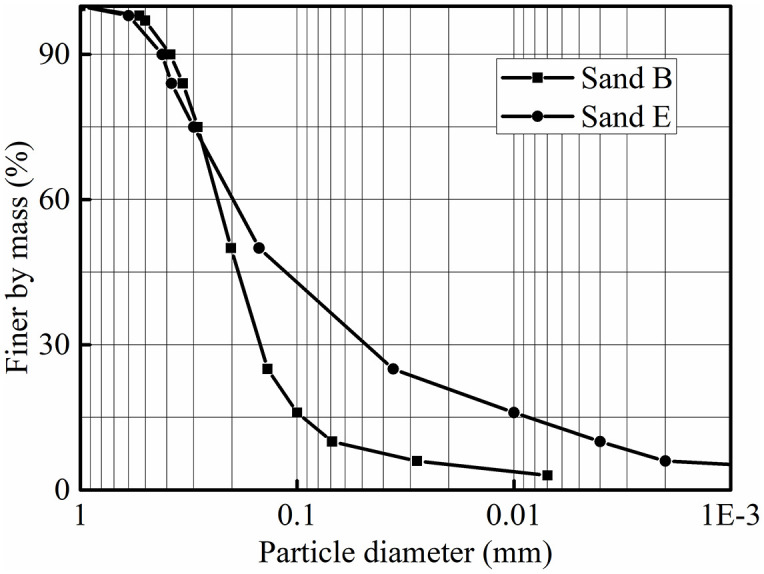
Grain size distributions of the experimental sands used in the tests.

The types of filling materials used in each project vary greatly, and there is no unified standard for them. As part of a coastal reclamation project in China, two types of sand (B and E) were selected for this study. Sand E was prepared by adding fine and coarse particles to sand B, resulting in a relatively wide gradation. Both samples were classified as continuously graded sand, and their grain size distributions are shown in Fig 4. The basic physical state properties of the two sand types are shown in Table 1.

**Table 1:** Basic physical state properties of the two sand types.

properties	Sand B	Sand E
d_10_, unit: (mm)	0.069	0.004
d_50_, unit: (mm)	0.202	0.15
d_90_, unit: (mm)	0.385	0.42
Coefficient of uniformity C_u_	3.4	50
Coefficient of curvature C_c_	1.5	3.1
Specific gravity G_s_, unit:(g/cm^3^)	2.65	2.65
Minimum porosity *n*_*min*_	0.29	0.33
Maximum dry density *ρ*_*d,max*_, unit:(g/cm^3^)	1.88	1.78

### 2.2. Determination of the flow velocity in the joint channel

To design the test scheme and control the test variables, it is necessary to determine the velocity of scouring flow in the channel. Currently, neither the engineering community nor academia has addressed this issue, and no detailed or reliable data are available for reference. The scouring flow in the channel mainly results from seepage along the sidewalls. Based on the position of the end of the channel and the downstream water level, the flow in the channel can be classified as either pressurized or free outflow, with a slight difference in the flow velocity. In the laboratory model tests, to ensure that the sand near the damaged area in the sand chamber was saturated and that the seepage process entailed saturated flow, this study focused only on submerged conditions with outflow pressure. On this basis, the flow velocity in the channel was approximately calculated via a hydraulic method, as described below.

The flow in the pressurized outflow channel originates from seepage through the surrounding sidewalls. It should be noted that the flow rate in the channel is equal to the seepage rate. The channel is approximated as a rectangular structure with a cross-sectional width *b* and total length *l*. The area of each sidewall is *b* × *l*, and the total area of the channel sidewalls is as follows.


$$\begin{array}{c} A_{s}=4bl\text{\textbackslash{}hspace\{0.33em}\}\left(\text{for\textbackslash{} four\textbackslash{} sidewalls}\right) \end{array}$$ (1)

where *A*_*s*_ is the total sidewalls area of the channel.

Seepage along the sidewall follows Darcy’s law, and the seepage rate is as follows.


$$\begin{array}{c} Q=kiA_{s}=4kbli \end{array}$$ (2)

where *k* is the seepage coefficient, and *i* is the seepage gradient.

The flow velocity in the channel can be obtained by dividing the channel seepage rate by the cross-sectional area of the channel.


$$\begin{array}{c} v=\frac{Q}{A_{c}}=\frac{Q}{b^{\text{2}}}=\frac{4lki}{b} \end{array}$$ (3)

where *A*_*c*_ is the cross-sectional area of the channel.

According to existing engineering data, a single-joint channel typically has dimensions of 0.05 m × 0.05 m × 10 m. That is, in the above formulas (1)-(3), *b* = 0.05m and *l* = 10m.

Field and laboratory tests conducted by Xu et al. [29] on seepage in the foundation of the eastern section of the Qingcaosha dam showed that the sand-filled geotextile tube has a seepage coefficient on the order of 10^–4^ cm/s. Based on this range, a relatively high seepage coefficient of *k* = 1 × 10^–3^ cm/s was adopted in this study to consider more unfavorable conditions. In addition, a conservative seepage gradient of *i* = 2 was used.

Substituting these values into Eqs. (2) and (3) yields the flow rate and flow velocity in the channel as follows.


$$\begin{array}{c} Q=40cm^{\text{3}}/s;\text{\textbackslash{}hspace\{0.33em}\}v=1.6cm/s \end{array}$$ (4)

Considering the differences between theoretical calculations and actual engineering projects, a range of velocities surrounding the calculated velocity was chosen. To separately examine the effects of seepage and scouring flow and to simplify the operation, the scouring flow velocity in the channel was set to five fixed values (0, 1, 2, 3, and 4 cm/s), rather than being varied with the seepage gradient or coefficient. Specifically, the desired scouring flow velocity was achieved by adjusting the height of the scouring flow tank, and the corresponding flow rate was monitored in real-time using a flow velocity meter installed on the joint channel. The relationship between tank height and flow velocity was pre-calibrated to ensure accurate setting of the five target values.

### 2.3. Test scheme and preparation

In this test, sand was filled via dry tamping, with the specific operation process reported in the literature [24]. This method aims to achieve the densest state of the sand specimens, i.e., a relative density (*D*_*r*_) of 1 for both sand types. In the filling process, piezometers were embedded at the positions shown in Fig 3. Before the beginning of each test, the flow velocity in the channel was set by adjusting the height of the scouring flow water tank, and the flow velocity was then maintained constant in the test. The seepage gradient was subsequently increased by incrementally increasing the height of the seepage flow water tank 1 cm at a time. Under the same head, piezometer readings and the flow rate at the outlet were observed every 1 min. When the piezometer readings and seepage rate remained unchanged for two consecutive minutes and if no obvious seepage channels were observed in the sidewall of the sand chamber or the sand surface, sand seepage was considered stable under this head, which was then maintained for 20 min before the head was increased by another 1 cm to continue the test. This procedure was continued until the soil sample failed completely.

## 3. Results and discussion

### 3.1. Failure mode of sand under seepage and scour

The experimental results showed that the scouring flow affects the sand transport rate in the joint channel but does not affect the failure mode or stage sequence of the sand in the sand chamber. That is, its influence is primarily on the time scale of the failure process rather than on the failure mode. Regardless of flow conditions, the failure process consistently exhibited three distinct stages based on changes in water turbidity and sand migration characteristics: a stable stage, an initial sand erosion stage, and a cyclic sand outflow stage. Although no abrupt changes were observed in the piezometric tube readings or seepage flow rate during the tests, these visual indicators proved sufficient to define the stage boundaries.

**Fig 5:**
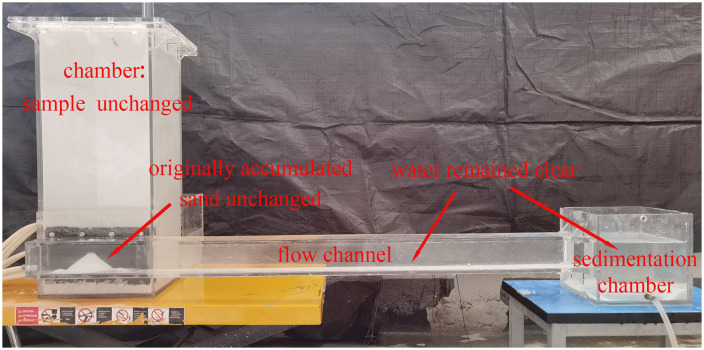
Stable stage in the test.

**Fig 6:**
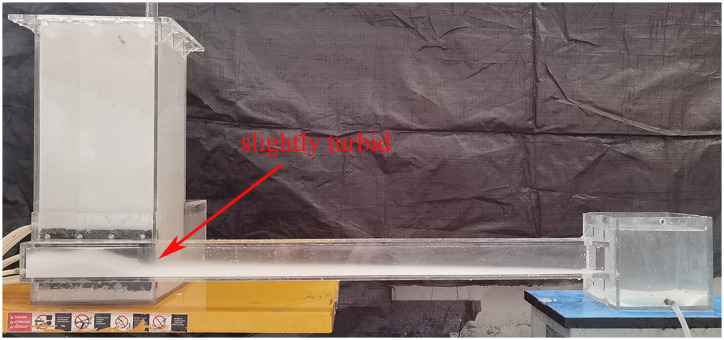
Incipient state of the initial sand outflow stage in the test.

**Fig 7:**
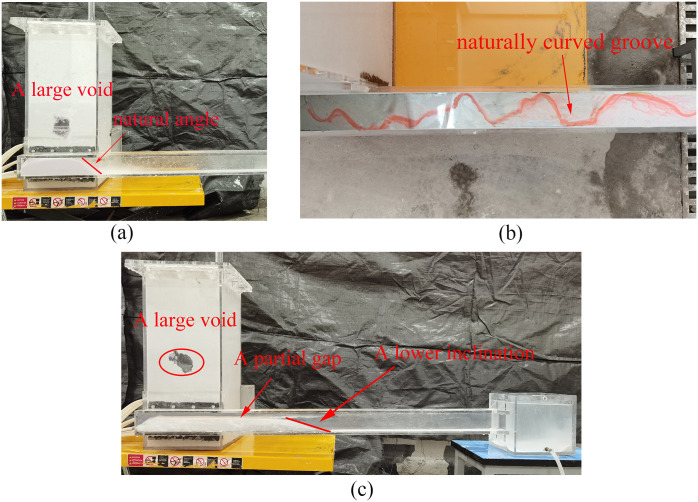
Final stable state in the test: (a) Front view of v = 0; (b) top view of v = 0; (c) front view of v > 0.

1. At the stable stage, with an incremental increase in the upstream hydraulic head, there was a gradual increase in the piezometric head and seepage rate. At this stage, there was no significant change in the sample in the sand chamber or any originally accumulated sand in the channel. The flow in the channel and the water in the downstream sedimentation chamber remained clear (as shown in Fig 5), indicating that the sand structure remained stable at this stage, and that the hydraulic gradient between the upstream and downstream regions did not reach the critical gradient for sand failure.

2. At the initial sand erosion stage, on the basis of the amount of sand outflow at the damaged area and the changes in the accumulated sand structure within the channel, this stage can be further divided into incipient, development, and quasi-steady state.

(1) In the incipient state of initial sand erosion, the sand that originally accumulated near the damaged area began to collapse and leak out of the damaged hole. Fine particles in the sand chamber began to flow out through the hole. These fine particles were carried in the water flow in the channel, causing the water in the channel to become slightly turbid, as shown in Fig 6.

(2) In the development state, compared with the loss of a few fine particles in the incipient state, sand particles of various sizes in the sand chamber near the damaged area began to flow out violently on a large scale and accumulate in the channel. With increasing amount of moving sand, sand started to migrate along the channel toward the sedimentation chamber. During the test, even under a low scouring flow velocity, sand could still flow out from the hole and migrate along the channel as long as the seepage gradient was high enough. Therefore, sand accumulation and migration in the channel were mainly the result of the seepage force in the sand chamber, which is not affected by the scouring flow velocity.
(3) In the quasi-steady state, as the sand in the channel gradually became compact and the accumulation length gradually increased, the support force of the sand in the channel on the sand in the damaged area gradually increased. When the support force exceeded the seepage force, seepage failure stopped, and the sand in the sand chamber no longer flow out. Then the water in the channel and the sedimentation chamber quickly became clear again.

3. In the cyclic sand outflow stage, with increasing hydraulic gradient, seepage failure occurred again, causing the sand in the sand chamber to move and the accumulated sand in the channel to continue migrating toward the sedimentation chamber. This continued until the accumulated sand in the channel lengthened and seepage failure stopped again, with the sand in the sand chamber and channel reaching stability again. Thereafter, each time the head was increased, the sand accumulated in the channel moved slightly more toward the sedimentation chamber. Owing to the obstruction of the accumulated sand in the channel, the sand in the sand chamber at each head level could reach a stable state after part of the sand was removed. The test entered a cycle of increasing the head—sand outflow—extension of accumulated sand—stopping sand outflow—increasing the head again. This process continued until the seepage rate increased instantly, which suggests that the sand structure in the sand chamber was completely destroyed. In this process, a large void may appear in the sand, as shown in Fig 7(a) and (c). This cyclic process is also unaffected by scouring flow, which again suggests that the sand flowing out of the damaged tube and the failure of the sand structure in the tube were caused mainly by seepage in the sand chamber.

Notably, when no flow was supplied from the scouring flow water tank, the flow velocity in the channel was negligible (approximately zero). Under this condition, the accumulated sand advanced across the full cross-section, with its downstream end forming a natural angle of repose, as shown in Fig 7 (a). Furthermore, observations through the channel’s top plate revealed that a naturally curved groove formed on the surface of the accumulated sand, which serves as seepage flow path. Owing to the extremely low flow rate, the flow path formed by the sand groove cannot be visually identified. To visualize the sand groove more clearly during testing, red ink was injected into the channel after the test. The ink traced the flow path of the groove, highlighting its meandering pattern within the channel (Fig 7 (b)).

When the scouring flow water tank supplied flow to the channel, the accumulated sand no longer filled the entire cross-section, resulting in a partial gap in the upper part. Under these conditions, the sand advanced in a semi-cross-sectional shape. Compared to the quasi-steady state under zero flow velocity (Fig 7 (a)), the inclination angle of the accumulated sand decreased significantly once a new equilibrium was reached, as shown in Fig 7 (c).

### 3.2. The critical gradient analysis

After the onset of the initial erosion stage, the internal structure of the soil is altered. Because the hydraulic gradient within the sand chamber is not uniform but increases as flow approaches the damaged hole [24], using the overall gradient between the upstream and downstream ends would underestimate the local gradient that actually drives sand erosion. To address this, the hydraulic gradient at the initial erosion stage, measured between the far-right piezometer (the one closest to the outlet) and the damaged hole, is defined as the critical hydraulic gradient (*J*_*cr*_). At this incipient state, since there is little sand in the joint channel and near the damaged hole, the water level at the outlet approximately equals the water head at the damaged area. Thus, the critical gradient *J*_*cr*_ is calculated via Eq. (5):


$$\begin{array}{c} J_{cr}=\frac{P_{\text{1}}-P_{\text{2}}}{L} \end{array}$$ (5)

where P₁ and P₂ are the hydraulic heads at the far-right piezometer and the outlet, respectively, and L is the horizontal distance between these two points. In this case, *L* = 3 cm (Fig 3). The critical gradient in the test under each condition is provided in Table 2

**Table 2:** Critical gradients of seepage failure under various conditions via Eq. (5).

	Flow velocity in the channel (cm/s)	Radius of the damaged area (cm)				
		0.25	0.5	1.0	1.5	2.0
Sand B	0	1.82	0.81	0.39	0.18	0.11
	1	1.61	0.70	0.32	0.14	0.07
	2	1.37	0.58	0.25	0.08	0.01
	3	1.06	0.43	0.17	0.02	/
	4	0.72	0.28	0.09	/	/
Sand E	0	3.15	1.65	0.94	0.65	0.52
	1	2.87	1.48	0.83	0.55	0.43
	2	2.44	1.24	0.68	0.44	0.33
	3	1.92	0.96	0.49	0.30	0.20
	4	1.33	0.64	0.31	0.14	0.06

Note:/ indicates that the sand structure in the geotextile tube failed when the seepage gradient was applied for the first time.

Table 2 indicates that the resistance of sand to seepage and scouring varies significantly with its gradation. Nevertheless, the influence of both damaged area and flow velocity follows a consistent trend: a larger damaged area coupled with a higher flow velocity leads to a pronounced reduction in resistance against seepage deformation. A detailed analysis of how each factor affects sand stability is presented in the following section.

#### 3.2.1. Effects of the grain size distribution and damaged area.

**Fig 8:**
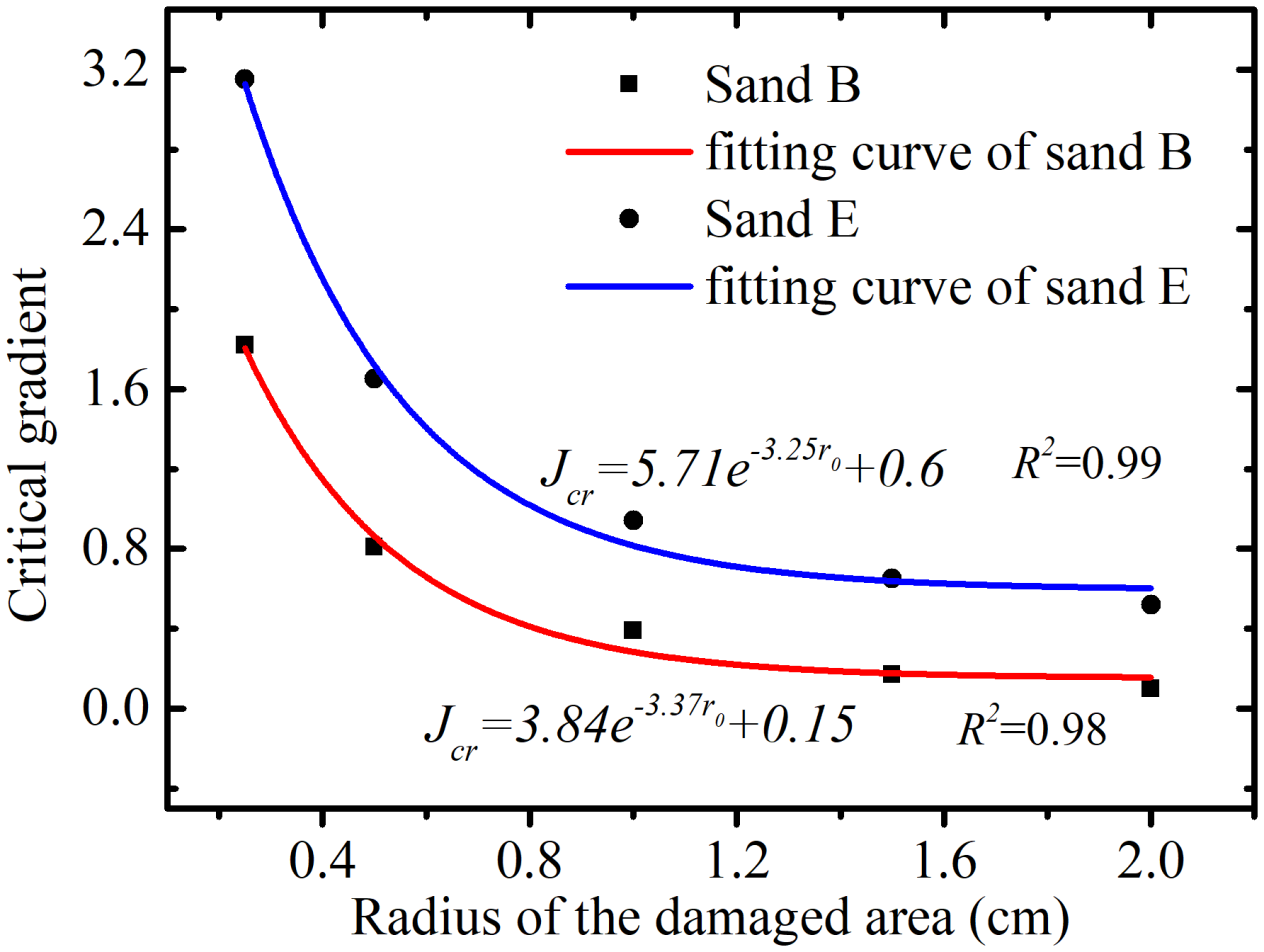
Variation in the critical gradient with the size of the damaged area in the test without scouring flow for two sand gradations.


To analyze the effects of the grain size distribution and damaged area on the seepage failure critical gradient, the scouring flow velocity was set to 0. The critical hydraulic gradient data under this condition were first plotted as a scatter plot and subsequently fitted with a nonlinear curve. Among the evaluated models, the exponential decay formula (Equation 6) demonstrated the best fit, as determined by the coefficient of determination. The critical gradient data, the fitted curve, and the corresponding *R²* are presented in the Fig 8.

$$\begin{array}{c} J_{cr}=ae^{-br_{\text{0}}}+c \end{array}$$ (6)

Where, a, b, and c are fitting constants, e is the Euler’s number. Theoretically, the critical gradient (*J*_*cr*_) of the sand within the geotextile tube approaches infinity when the hole radius (r₀) is zero. However, the (a + c) values calculated from Equation (6) are 3.99 and 6.31 for the two sands, respectively, which are inconsistent with the theoretical ideal. Therefore, this model is recommended for predicting the seepage stability of sand specifically in damaged tubes. As the damaged radius becomes sufficiently large, *J*_*cr*_ asymptotically approaches the constant c. Thus, c represents the inherent critical gradient of the sand material,

As shown in Fig 8, the critical gradient decreases as the damaged area increases. This trend occurs because a larger damaged area results in greater exposure of the sand, thereby reducing the geotextile’s capacity to protect and restrain the soil. This conclusion is consistent with findings reported in the literature [10,11]. From the perspective of Terzaghi’s soil-arching theory, a smaller hole not only reduces the arch span—thereby enhancing arch stability—but also minimizes deformation in the surrounding geotextile. This reduced deformation provides more stable abutments, which further promotes the development and maintenance of the soil arch [30].

When the damaged radius exceeds 2 cm, the curve plateaus, and the critical gradient converges to the constant c. This suggests that for sand materials similar to those used in this study, damaged dimensions larger than 2 cm should be avoided in practical applications. Should they occur, immediate remediation is required to restore functionality.

Fig 8 also shows that Sand E exhibited a significantly greater critical gradient than that of sand B. Further calculations based on Table 2 show that when the scouring flow velocity increases from 0 to 4 cm/s, as the damage radius increases, the critical gradient of Sand B decreases by 94%–100%, while that of Sand E decreases by 83%–95%, indicating that the damage radius has a relatively smaller effect on Sand E. These findings confirm that grain size distribution significantly influences the seepage stability of damaged tubes, which is fully consistent with established conclusions in prior research on soil seepage stability [31].

The gradation curves show that the coefficient of uniformity (*C*_u_) is 3.4 for sand B and 50 for sand E. According to the criteria of Istomina [32], sand B is an internally stable soil, whereas sand E is an internally unstable soil. Thus, the critical gradient of sand E should theoretically be lower than that of sand B. If the criteria of Kezdi [33]and Kenney [34]were applied, similar conclusions would be drawn. However, this is inconsistent with the results of this test, indicating that the above criteria are not suitable for evaluating the seepage stability of sand-filled tubes.

Based on Terzaghi’s soil arching theory [30], the formation of soil arch structures relies on effective force chain transmission between particles. As demonstrated by Chen et al. [35] at the mesoscopic scale, fine particles tend to migrate into pore constrictions during seepage, causing localized clogging and altering the local pore structure. Applying this mechanism to the present study, Sand E contains a relatively high proportion of fine-grained particles, which facilitates such migration and clogging. This clogging reduces the effective pore size, increases inter-particle contacts, and enhances local stress redistribution, thereby promoting the development of a more stable soil arch structure and allowing for larger arch spans. Consequently, Sand E exhibits a higher critical hydraulic gradient than Sand B, which contains fewer fines and experiences less clogging.

Liu [36] divided soil into coarse and fine fractions in the study of the seepage stability of well-graded soil, with the dividing point being the geometric mean particle size, and then proposed the concept of an optimal fine-particle content on the basis of the porosity. The closer the fine-particle content is to the optimal fine-particle content, the greater the resistance of the sand to seepage. According to this criterion, the fine-particle content of sand E is closer to the optimal fine-particle content than that of sand B, and it therefore exhibits a greater resistance to seepage, which is consistent with the test results in the present study.

In addition, Li [37] used a fluid‒solid coupling model to numerically simulate the seepage failure process of soil and concluded that a wide particle size distribution corresponds to a high critical hydraulic gradient. These findings are consistent with the results of the present study.

Therefore, to ensure the seepage stability of geotextile tubes in the filling process, fill material with a wide gradation range and an optimal fine particle content is more beneficial for the stability of the geotextile tube structure.

#### 3.2.2. Effect of scouring flow.

The test results revealed that scouring flow greatly affects the critical gradient of seepage failure. With different gradations and damaged area sizes, the effect of the scouring flow velocity on the critical gradient varies. The effect of scouring flow on the geotextile tube structure is analyzed in detail from the perspectives of the damage size and particle gradation.

(1) Damage size

**Fig 9:**
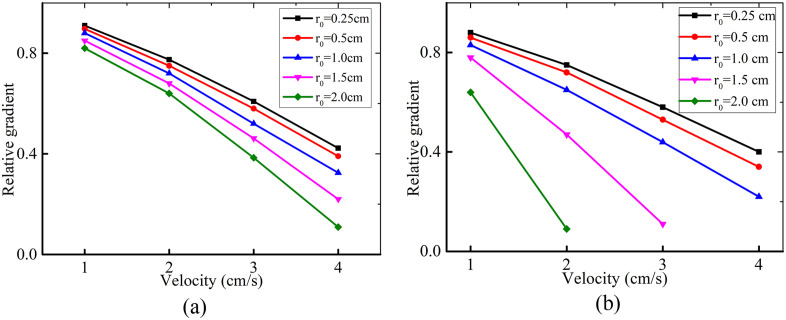
Variations in the relative gradient with scouring flow velocity under different damaged size: (a) Sand E, (b) sand B.

To analyze the effect of the scouring flow velocity on the critical gradient, the critical gradient under no scouring flow was used as the baseline, and the relative gradient was calculated as the ratio of the critical gradient with scouring flow to the baseline. The variation in the relative gradient as a function of the flow velocity is shown in Fig 9.

The figures show that scouring flow in the channel significantly affected the critical gradient of the sand in the sand chamber. The higher the scouring flow velocity in the channel is, the lower the critical gradient for sand seepage failure in the sand chamber. This is because when the scouring flow velocity in the channel was low, scouring flow did not reach the incipient flow velocity of the majority of the sand particles, so the flow exerted a negligible effect on sand accumulation in the channel. When the flow velocity was high, sand particles began to move under the action of scouring flow, causing structural failure of the sand near the damaged area, which in turn led to a decrease in the critical gradient. This theory has been supported by many studies in the field of sediment dynamics [38,39].

With increasing flow velocity, the decrease in the critical gradient accelerated. When the scouring flow velocity in the channel was low, the critical gradient decreased slowly with increasing scouring flow, but as the flow velocity was increased, the rate of decrease of the critical gradient increased rapidly. This can be explained from the perspective of sediment incipient motion. Among the many factors influencing sediment incipient motion, the flow velocity plays a critical role as an external factor [19]. Many studies have proposed various formulas for the flow velocity of sediment incipient motion [40,41]. Although these formulas vary in form, the effect of the flow velocity on sediment incipient motion is not linear but rather follows an exponential or logarithmic distribution. On the other hand, to achieve sediment incipient motion, the flow affect the sediment, namely, it must consume a certain amount of kinetic energy, which is proportional to the square of the velocity [42]. Bagnold [43] also reported that the incipient power is proportional to the 3/2 power of the particle size. In this experiment, the effect of velocity on the critical gradient was also not linear. As the scouring flow velocity increased, the rate of decrease of the critical gradient increased rapidly.

A further comparison of the curves revealed that the slope of the curve was low when the radius was smaller than 1 cm, and that the slope of the curve increased significantly when the radius was greater than 1 cm. This result indicated that the larger the damage size is, the greater the effect of the scouring flow velocity on the critical gradient. This occurs because a larger damage size leads to a larger scoured area of sand within the damaged area, thus affecting more sand particles and causing a more significant adjustment of the sand structure in the sand chamber.

(2) Sand gradation

**Fig 10:**
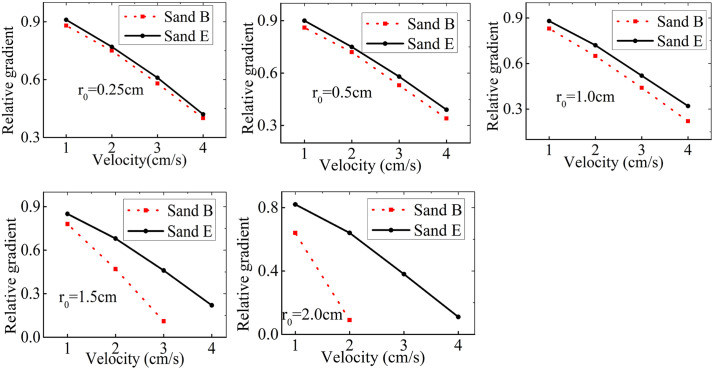
Variations in the relative gradient with scouring flow velocity under different sand gradations.

Under the different sand gradations, scouring flow affected the critical gradient to varying degrees. Fig 10 shows the variation trends of the relative gradient with scouring flow velocity under the same damage size but for different sand gradations. As shown in the figure, the relative gradient of sand B was consistently lower than that of sand E under the same working conditions, indicating that the structural strength of sand B geotextile tubes was more significantly affected by scouring flow. Calculations based on Table 2 show that when the damage radius increases from 0.25 cm to 2.0 cm, as the scouring flow velocity increases, the critical gradient of Sand B decreases by 60%–100%, while that of Sand E decreases by 58%–88%, further confirming the same conclusion.

This occurs because sand E contained more fine particles, which enabled it to exhibit a certain degree of structure. Fine particles were more likely to be caught in the voids between large particles tying the particles together and thus making it difficult for them to undergo incipient motion from scouring flow, whereas coarse particles with no fines were more exposed and easier to mobilize [19]. In addition, according to the Shields [44] curve, when the sand particle diameter is smaller than 0.1 mm, the incipient flow velocity increases with decreasing particle diameter. According to the particle gradation curve of sand E, its fine sand particles smaller than 0.1 mm accounted for nearly 50% of the total mass. That is, comparatively, sand B contains more mobile particles, so sand E was less affected by the flow velocity under the action of scouring flow. This is basically consistent with the research findings of Dou [45] on the incipient motion of sand particles under the action of a scouring flow.

**Fig 11:**
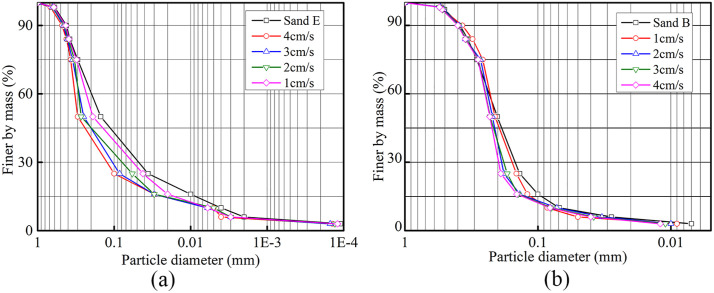
Effect of Flow Velocity on Gradation Change.

To validate the above analysis, Sand B and Sand E were collected from the joint channel after the test, and their grain size distributions are shown in the Fig 11. Fig 11 reveals that higher flow velocities lead to more pronounced coarsening. A comparison between Figures (a) and (b) reveals that the degree of coarsening is significantly greater for Sand E than for Sand B. Furthermore, the change in Sand E is primarily limited to fine particles between 0.01 and 0.2 mm in diameter. Coarse particles and fine particles below 0.01 mm show little change. This finding supports the previous conclusion that fine particles (particularly those <0.01 mm) are not easily transported, which is attributed to clogging effects and enhanced inter-particle contacts.

Fig 10 further indicates that with increasing damage size, the difference in the relative gradient between the two types of sand gradually increased. This trend suggests that the degree of influence of scouring flow on the critical gradient is related to the size of the damaged area. The larger the radius of the damaged area is, the more significant the effect of scouring flow. This mainly occurs because the size of the damaged area determines the contact area between the scouring flow and the sand. Relevant analysis has already been provided in detail earlier and therefore is not repeated here.

## 4. Conclusion

In this study, a set of model tests were conducted to provide a detailed description of the erosion progress and failure mechanism of soils in damaged geotextile tube subject to seepage and scouring. The following conclusions can be derived based on the results obtained from this study:

1. Stable, initial erosion and cyclic sand outflow are the three stages in the sand seepage and scouring failure process. The failure mode and the failure stages sequence of sand in the sand chamber are not affected by whether scouring flow is applied in the joint channel. However, scouring flow affects the shape of the accumulated sand and the form of its migration. When the scouring flow is absent, the sand moves forward in the full cross-section within the channel, with irregular grooves formed at the top of the accumulated sand and the end of the accumulated sand reaching its natural angle of repose. When the scouring flow is present, the sand moves forward in half-section within the channel, with a flatter inclination angle at the downstream end.
2. The critical hydraulic gradient, which represents the seepage stability of the soil, corresponds to the initial erosion stage. To ensure the seepage stability of geotextile tubes, Liu’s method of determining the optimum fine particle amount in sand-filled geotextile tubes provides greater stability than other methods do. This suggests that the fill material in engineering should exhibit a wide gradation range and the optimal fine particle content.
3. An exponential decay model was established to describe the relationship between the critical gradient and the damaged area. When the damaged radius exceeds 2 cm, the critical gradient remains constant. This implies that, for the sand material used in this study (or sand with a similar particle size distribution), the damaged radius in geotextile tubes should not exceed 2 cm in practical engineering.
4. The effect of the scouring flow velocity on the critical gradient is not linear. Notably, with increasing flow velocity, the critical gradient decreases exponentially. For the same sand gradation, the larger the damage size is, the greater the effect of the scouring flow velocity on the structural integrity of the geotextile tubes. When the damaged area remains constant, well-graded sand with more fine particles is more resistant to scouring than poorly graded sand with more mobile particles is.

### 4.1. Future research

Conclusions drawn in this study elucidate the soil failure patterns and influencing factors of sand loss through damaged hole and joint channels. These conclusions can provide a valuable reference for the design, construction, and durability protection of geotextile tubes. However, the current research has not yet established a stability criterion or assessment method that considers the combined effects of multiple factors. It also does not account for the influence of the geotextile properties, such as porosity and thickness, or the gravel grain size and content within the sand matrix. In subsequent research, the authors will incorporate these various factors and employ statistical methods to develop a comprehensive, multi-factor stability criterion.

### 4.2. Notation

Basic SI units are given in parentheses.
*b* cross-sectional side length of the joint channel (m)
*l* total length of the joint channel (m)
*A*_*s*_ total area of the channel sidewalls (m^2^)
*A*_*c*_ cross-sectional area of the channel (m^2^)
*i* seepage gradient (dimensionless)
*k* seepage coefficient (dimensionless)
*Q* seepage rate (m^3^/s)
*v* velocity of water in the channel (m/s)
*d*_*10*_ grain diameter at which 10% of the soil particles (by weight) are finer. (mm)
*d*_*50*_ grain diameter at which 50% of the soil particles (by weight) are finer. (mm)
*d*_*90*_ grain diameter at which 90% of the soil particles (by weight) are finer. (mm)
Gs Specific gravity (g/cm3)
*n*_*min*_ Minimum porosity
*ρ*_*d,max*_ Maximum dry densit (g/cm3)
*D*_*r*_ relative density
*P*_*1*_ hydraulic heads at the far-right piezometer
*P₂* hydraulic heads at the outlet
*L* horizontal distance between the piezometer on the far right and the joint channel (m)
*j*_*cr*_ critical gradient (dimensionless)
*C*_*u*_ coefficient of uniformity (dimensionless)
C_c_ coefficient of curvature (dimensionless)
*r*_*0*_ radius of the damaged area (m)

## Supporting information

S1 File Data release. All data addressed in this manuscript is available in the supplemental file Data_release.
(XLSX)
